# Inline Determination of Residence Time Distribution in Hot-Melt-Extrusion

**DOI:** 10.3390/pharmaceutics10020049

**Published:** 2018-04-15

**Authors:** Jens Wesholowski, Andreas Berghaus, Markus Thommes

**Affiliations:** 1 Institute of Solids Process Engineering, TU Dortmund University, Dortmund 44227, Germany; jens.wesholowski@tu-dortmund.de; 2 ColVisTec AG, Berlin 12489, Germany; a.berghaus@colvistec.de

**Keywords:** UV/Vis spectroscopy, Residence Time Distribution, twin screw extrusion, process analytical tool, Quality-by-Design

## Abstract

In the framework of Quality-by-Design (QbD), the inline determination of process parameters or quality attributes of a product using sufficient process analytical technology (PAT) is a center piece for the establishment of continuous processes as a standard pharmaceutical technology. In this context, Twin-Screw-Extrusion (TSE) processes, such as Hot-Melt-Extrusion (HME), are one key aspect of current research. The main benefit of this process technology is the combination of different unit operations. Several of these sub-processes are linked to the Residence Time Distribution (RTD) of the material within the apparatus. In this study a UV/Vis spectrophotometer from ColVisTec was tested regarding the suitability for the inline determination of the RTD of an HME process. Two different measuring positions within a co-rotating Twin-Screw-Extruder were compared to an offline HPLC–UV as reference method. The obtained results were overall in good agreement and therefore the inline UV/Vis spectrophotometer is suitable for the determination of the RTD in TSE. An influence of the measuring position on repeatability was found and has to be taken into consideration for the implementation of PATs. An effect of the required amount of marker on process rheology is not likely due to the low Limit-of-Quantification (LoQ).

## 1. Introduction

In recent years, the pharmaceutical industry has had to deal with challenges related to the concept of Quality-by-Design (QbD) [1] and a shift of production focus from batch to continuous manufacturing. A major benefit is a potential decrease of fluctuations in product quality. Therefore, the determination and characterization of critical process parameters is crucial.

**Figure 1:**
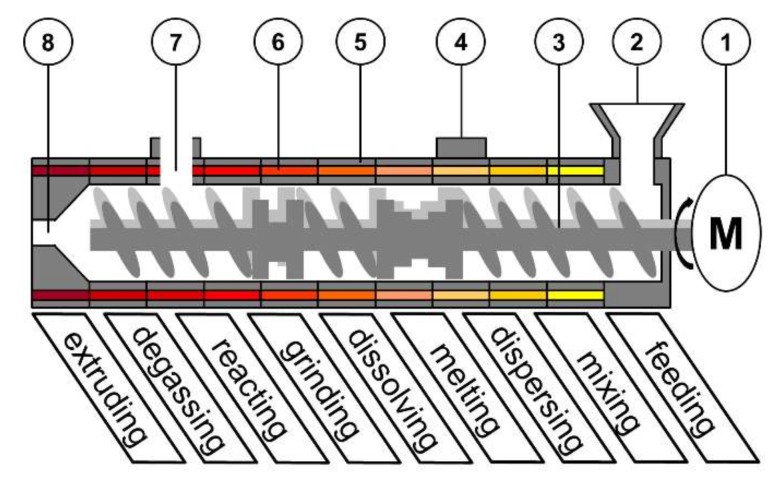
Schematic set-up of a co-rotating Twin-Screw-Extruder: (**1**) motor; (**2**) feed hopper; (**3**) screws; (**4**) port; (**5**) barrel; (**6**) barrel heating; (**7**) degassing port; (**8**) die.

In this context Twin-Screw-Extrusion (TSE, Figure 1) has been a focus of research interest. A typical application for this continuous, robust and cost-efficient process is the production of solid dispersions by Hot-Melt-Extrusion (HME) [2,3], where an active pharmaceutical ingredient (API) is dissolved and distributed within a molten matrix carrier.

In such a process, various unit operations are combined in one step. Mechanism feeding, degassing and extruding have a crucial impact on the product homogeneity, uniformity and shape [4,5,6,7]. However, these are mainly driven by the equipment. The sub-processes of melting, mixing, dispersing, reacting, grinding and dissolving, are dependent on different process parameters (e.g., feed load, temperature, specific mechanical energy, shear stress) and combinations of those [8,9,10,11]. The screw configuration is also a crucial variable on different sub–processes, e.g., for solvent–free salt preparation during extrusion, in combination with temperature [12].

**Figure 2:**
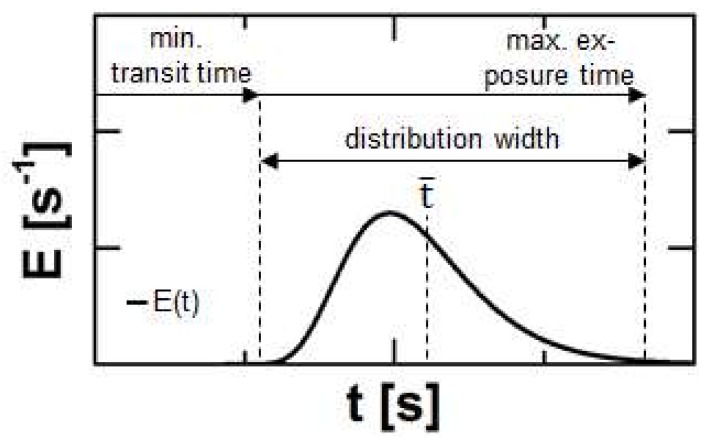
Typical course of a residence time density function for Twin-Screw-Extrusion and process related information regarding to certain sections or single points of this curve.

A parameter reflecting information on all these mechanisms is the Residence Time Distribution (RTD), typically expressed by the residence time density function E (Figure 2). The experimental determination is performed either as a pulse experiment by adding a marker substance as Dirac impulse or as step experiment by an instant change of the processed formulation. In this case, the integrated or cumulative residence time density function F is obtained.

The onset of a typical RTD plot defines the minimal transportation time of the material through the extruder and is linked to the minimum duration for melting, reacting, dissolving or grinding and is an indicator e.g., for crystal residues. The width of the distribution contains information about the flow regime. A tighter RTD is connected to plug flow and therefore reduced distributive mixing, while a broad RTD refers to a high axial dispersion of the material [13]. The medium residence time t ¯ represents the average transit time and respective mean processing time. The offset of the RTD highlights the maximum exposure time to mechanical and thermal stress. This correlates to degradation processes [14,15].

Many process analytical techniques (PAT) are already available for the RTD determination of TSE processes. These are classified into offline and inline methods. Offline techniques include e.g., the determination of colour intensity [16], radioactive radiation [17] or UV-absorption [18]. However, this type of technique leads to a shift between actual process and measured time. This is contrary to the idea of QbD, since the product quality should be controlled in real time. Therefore, inline measurement devices are necessary. For a few techniques, e.g., determination of magnetic susceptibility [19] or light scattering [20], the sensitivity regarding the necessary amount of marker remains unclear. The content of such an additive affects the rheology of the process and the tracer content should remain as low as possible. This prerequisite is fulfilled for a lately developed inline video analysis [21], which has a resolution of 0.03 wt % of added marker to the mass flow per minute. UV–fluorescence spectroscopy for RTD determination [22] also has a high potential regarding a minimized marker content, since the resolution is up to 0.001 wt %. These are not standard applications for HME in comparison to spectroscopic methods like NIR- or Raman-spectroscopy [23]. These have already been successfully implemented. However, for those techniques a Dirac–impulse of a few percent of the marker has to be added to the process. Therefore, these methods do not seem to be ideal for RTD determination.

The aim of this study is to investigate the suitability of an inline UV/Vis-spectroscopy device for the RTD determination of an HME process. Here one major objective is the validation of the obtained inline data by an offline reference. Moreover, the limit of quantification according to the ICH Q2 methodology [24,25] is revealed. This parameter correlates with the necessary tracer amount during experimental investigations of the RTD and is a suitable objective for the comparison between different techniques.

## 2. Materials and Methods

### 2.1. Hot-Melt-Extrusion on a Co-Rotating Twin-Screw-Extruder

**Figure 3:**
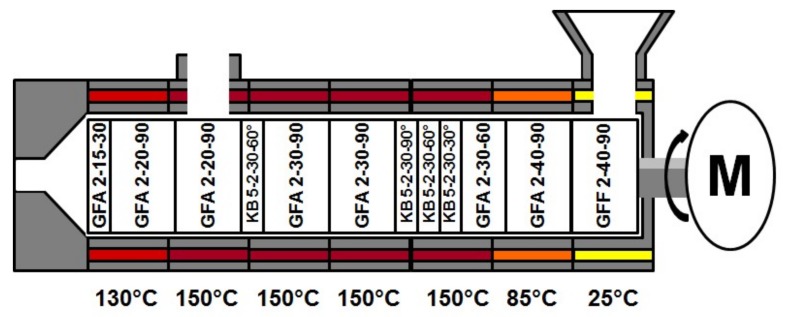
Screw configuration and barrel temperature profile for conducted Hot-Melt-Extrusion experiments. GFA: conveying; KB: kneading elements; GFF: conveying elements with an enlarged free volume.

For the extrusion experiments polyvinylpyrrolidone vinylacetate (copovidone; Kollidon VA 64, BASF, Ludwigshafen, Germany) served as model substance and a gravimetric feeder (KT-20, K-Tron, Niederlenz, Scwitzerland) was used. For the RTD determination a 100 mg of quinine dihydrochlorid (Caesar & Loretz, Hilden, Germany) was utilized as marker substance and fed directly into the Twin-Screw-Extruder (Leistritz 27 GL 28 D, Nurenberg, Germany). Experiments were performed for a screw speed of 100 rpm, at a feed rate of 30 g·min^−1^, with a constant screw configuration and barrel temperature profile (Figure 3) and carried out in triplicate. The common screw configuration [18] basically consisted of conveying (GFA) and kneading elements (KB), while at the feed port special conveying elements with an enlarged free volume (GFF) were used. Regarding the conveying elements, the numbers on the figure encode for numbers of flights, pitch length, element length respective to number of discs, number of flights, element length, and stagger angle for the kneading elements. For one run of the three repetitions the RTD was determined inline simultaneously at two positions, while samples were collected for an offline analysis with a reference method.

### 2.2. Offline Determination: HPLC-UV

Samples were collected for the offline measurements in varying rates (0.03 to 0.1 Hz) over 10 min, dissolved in methanol (Rotisolv HPLC gradient grade, Carl Roth, Karlsruhe, Germany) and analysed by a combination of HPLC (2695 Separations Module, Waters, Milford, MA, USA) and UV/Vis spectroscope (2487 Dual λ Absorbance Detector, Waters, Milford, MA, USA) at a volume flow of 1 mL/min of methanol as eluent. The column (LiCrospher 100 Rp-18 endcapped (5 µm) LiChroCart 125–4, Merck KGaA, Darmstadt, Germany) temperature was 45 °C. The marker was detected at 330 nm, the integrated signal over time is directly correlated to its concentration. Each data set is nominated by the integral value over all considered sampling points of the corresponding data set.

### 2.3. Inline Determination: UV/Vis Spectroscopy

**Figure 4:**
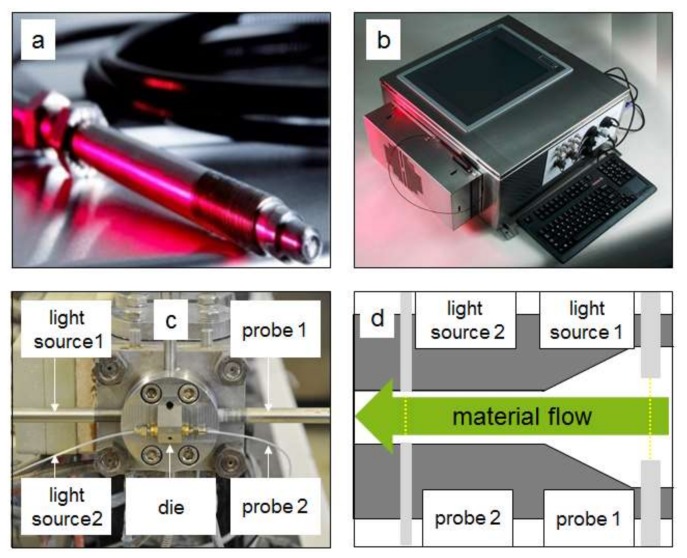
Inline measurement system based on UV/Vis spectroscopy from ColVisTec for Twin-Screw-Extrusion (TSE): (**a**) inline probe; (**b**) UV/Vis spectrophotometer; (**c**) attached probes; (**d**) measurement positions.

For the inline measurement a device from ColVisTec (Inspectro X, ColVisTec, Berlin, Germany) was used. The measurement was executed with two probes (TPMP, ColVisTec, Berlin, Germany) in transmission, using a range of 220 to 800 nm and a sampling rate of 0.185 Hz. Two measurement positions were tested (Figure 4); position one was located directly behind the screw tips (POS 1) and the second position was in the die channel (POS 2). The second measurement position was sterically hindered, as free space for mounting the probes was minimal. Therefore, single fibres were attached. The detection at the two different positions was executed simultaneously.

## 3. Results & Discussion

### 3.1. Data

**Figure 5:**
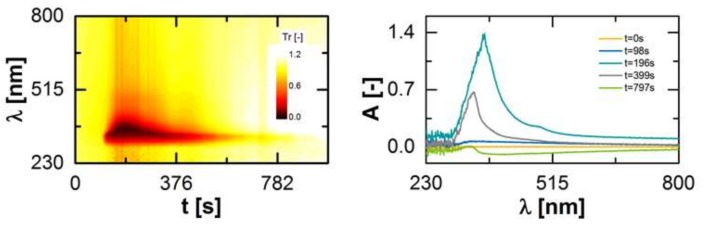
Detected marker signal at the extruder outlet over time as a function of the wavelength (**left**). Measurement signal transformed to an absorbance *A* for different time steps after the marker substance was added (*t* = 0 s; (**right**)).

The RTD was determined by adding a marker substance in the hopper of the extruder and measuring the Transmission *T*r (Equation (1)) as response signal with respect to time *t* and the wavelength λ (Figure 5, left) at the die in two different measuring positions (Section 2.3).

(1) $$Tr\left(\lambda ,\;t\right)=\frac{I\left(\lambda ,t\right)}{I_{0}\left(\lambda ,t\right)}$$

In accordance to the Lambert–Beer law, the transmission was converted for subsequent time steps into an absorbance *A* (Figure 5, right), which is a function of the marker concentration *c*, the path length of the light *d*_path_ and the absorbance coefficient of the marker ε_λ_.

*A*(λ,t) = −log_10_(*T*r(λ,*t*)) = *c*_marker_(*t*)·*d*_path_·ε_λ_(λ) (2)

Comparing the first (*t* = 0 s) and the last (*t* = 797 s) presented spectra, a shift of the baseline is visible. This is related to a change of the optical properties of the pure polymer melt in form of a higher transparency, e.g., due to fluctuations of the barrel temperature. This effect was considered within the data evaluation by a baseline correction over time.

For the determination of the residence time density function, *E*, an integrated value for a specific wavelength range of the absorbance spectra was calculated. This is necessary, since the absorption activity of the marker is located in the range of 300 to 800 nm and the peak is not located at the same wavelength over time. Then the integrated values were normalized by the integral of these over time, since the characteristic properties of residence time density function is an integral value of 1.

(3) $$E\left(t\right)=\frac{\int_{300\;\mathrm{nm}}^{800\;\mathrm{nm}}A\left(\lambda ,\;t\right)\;d\lambda }{\int \left(\int_{300\;\mathrm{nm}}^{800\;\mathrm{nm}}A\left(\lambda ,\;t\right)\;d\lambda \right)dt}$$

The integrals were calculated by the trapezoidal method. The obtained residence time density functions are characteristic for a process and can be utilized to directly compare different determination methods or measuring positions.

### 3.2. Comparison of Offline and Inline RTD Determination

**Figure 6:**
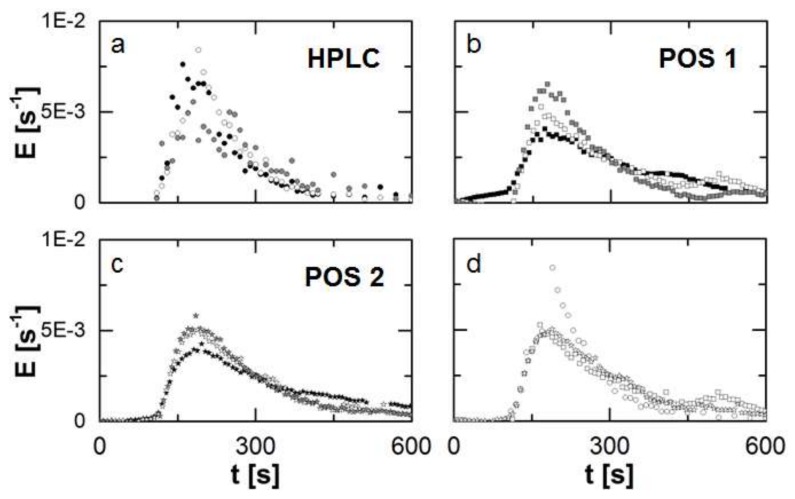
Obtained residence time density function (*n* = 3) for the different methods and positions (**a**)–(**c**) represented by the different symbols. The methods and positions are compared according to the detected Residence Time Distribution (RTD) by these for the second repetition of the experiments (**d**) as an example. Generally, each repetition is highlighted by a different colour.

The RTD of the TSE process was measured in triplicate for constant process parameters (screw speed, mass flow) and screw configuration. For each run the determination was executed simultaneously at two inline positions (POS 1: directly after the screws; POS 2: in the die flow channel), while the sample collection for an offlline determination by HPLC-UV. The samples were collected for 600 s and the same period of the recorded inline data is considered. From the recorded data sets discretized residence time density functions are obtained. The results of each of the three runs are presented for the individual measuring positions and methods (Figure 6).

For the offline determination (Figure 6a) a broader scattering of the single data-points is observed due to typical drawbacks of manual sampling, e.g., temporal fluctuations regarding the moment of sample collection or handling issues due to the high viscosity and temperature of the melt.

For the inline determination in POS 1 (Figure 6b) an appearance of a second peak in each run is observed. This is not typical for TSE processes and might be related to the attachment of the probes and an irregularity of the flow pattern.

In comparison to the other methods, repeatability is highest for the inline determination of the RTD in POS 2 (Figure 6c). The course of the curve is depicted clearly, fluctuations are minimized in comparison and no bimodal distribution is found.

A comparison of the determined RTD’s by the individual methods and measuring positions for the same run reveals an overall sufficient agreement among these (onset, peak and offset). This is classically presented in RUN 2 (Figure 6d). In accordance to the descriptive statistics, quantiles can be used to describe such a distribution (Table 1). In the case of an RTD the paramter *t*_i_ symbolizes a threshold in time for the ejection of *i*-wt% of the injected marker mass. This is expressed by a value of i100 for the cummulative residence time density function.

(4) $$\int_{t=0s}^{t_{i}}E\left(t\right)dt=\frac{i}{100}$$

The calculated quantiles are always the lowest for the offline determination. The calculation of the integral value (Equation (4)) was executed by the trapezoidal rule, which directly related to the overall resolution of the distribution in terms of the number of data points. A higher sampling rate lead to a smaller deviation from the true value and to a reduced underestimation of the real integral value. This effect is amplified for increasing quantile-values. The deviation between offline and inline determination was negligible for *t*_10_, while the difference increased from *t*_50_ to *t*_90_.

### 3.3. Suitability for RTD Monitoring

For the monitoring of an HME process the RTD can be used in order to obtain information about the several sub-processes (Figure 1 and Figure 2). Therefore experimental data can be fit by a RTD-model. Lately introduced was the Twin-Dispersion Model (TD model) [18], which is based on the convolution of two residence time density functions *E*_AD_ (Equation (5)) of the Axial-Dispersion Model (AD model).

(5) $$c\left(t\right)=\frac{c_{0,\;\mathrm{TD}}}{AUC}\overset{\infty }{\underset{-\infty }{\int }}E{\left(s,\;{\bar{t}}_{1},\;Bo_{1}\right)}_{\mathrm{AD}}\cdot E{\left(t-s,\;{\bar{t}}_{2},\;Bo_{2}\right)}_{\mathrm{AD}}ds\;=c_{0,\;\mathrm{TD}}\cdot E{\left(t\right)}_{\mathrm{TD}}$$

*AUC* represents the integral of the convolution for *t* in the range of 0 to ∞. An additional scalling paramter *c*_0_ is symbolizes the initial tracer concentration and *E*_AD_ represents two independent mixing processes. For these the mean residence time $\bar{t}$ is considered as well as the Bodenstein number *B*o, which describes the ratio of axial transport to dispersion. A higher *B*o-value is related to physically reduced mixing due to plug flow. The AD-model distinguishes between *B*o ≤ 100 (Equation (6)) and *B*o > 100 (Equation (7)).

(6) $$E{\left(t\right)}_{\mathrm{AD}}=\frac{1}{2\cdot \bar{t}}\cdot {\left(\frac{Bo}{\pi \;\cdot }\right)}^{0.5}\cdot e^{-\frac{Bo\cdot {\left(1-\theta \right)}^{2}}{4\cdot \theta }}\;$$

(7) $$E{\left(t\right)}_{\mathrm{AD}}=\frac{1}{2\cdot \bar{t}}\cdot {\left(\frac{Bo}{\;\pi \;}\right)}^{0.5}\cdot e^{-\frac{Bo\cdot {\left(1-\theta \right)}^{2}}{4}}$$

The TD-model was fit to the data sets by the least square method. The obtained characteristic parameters are given in Table 2.

For all methods and measuring positions *c*_0_ was close to 1 with a high repeatability (*cv* < 2%), since the data sets were nominated (Equation (3)) in order to obtain discrete residence time density functions. The repeatability for the inline determination in POS 2 was enhanced, indicated by the lowest *cv*-values for all characteristic parameters in comparision to the other tested methods. While the deviations are below 14% for ${\bar{t}}_{1}$ and ${\bar{t}}_{2}$ at this measuring position, the results are insufficient for *B*o_1_ and *B*o_2_. However, this is an issue related to process stability rather than the determination method. The curve of the detected discrete function for one run does significantly differ from the other two repetitions (Figure 6). Since *B*o_1_ and *B*o_2_ define the shape of the model function, this leads to the deviations for these fitted characteristic parameters.

### 3.4. Determination of the Limit of Quantification

The sensitivity of an applied method for RTD determination defines the required amount of marker and is characterized by the Limit of Quantification (LoQ). This parameter was based on the signal to noise ratio for the basic signal, the pure polymer melt. The mean value of the basic signal ${\bar{x}}_{\mathrm{basic}}$ was considered, as well as the corresponding standard deviation *s*_basic_. Due to the ratio of initial marker mass m_marker_ and the massflow of the total powder inlet  m ˙, the LoQ can be expressed as weight fraction of the marker within the extrudate.

(8) $$\mathrm{LoQ}=\frac{m_{\mathrm{marker}}}{\dot{m}}\;\left({\bar{x}}_{\mathrm{basic}}\;+10{\;s}_{\mathrm{basic}}\right)$$

The LoQ was only presented for the inline determination in POS 2, since this method prooved to be the most sufficient of the tested ones. Moreover, s was calculated based on the first 10 values (Figure 6) of a RTD experiment for each run. The determined LoQ with 87 ± 22 ppm proved the high potential of the used inline UV/Vis spectroscopy. This limit could be further reduced by a different marker with a higher signal strength. The reduced path length of the light beam in this measuring position has to be taken into account.

## 4. Conclusions

An inline UV/Vis spectrophotometer from ColVisTec was evaluated regarding its suitability for the determination of the RTD as a critical process parameter of HME. Therefore, a combination of HPLC and UV/Vis-spectroscopy was utilized as offline reference method. Experiments were carried out on a co-rotating lab-scale Twin-Screw-Extruder from Leistritz.

The data revealed a sufficient agreement between both methods, and repeatability was shown for each method. The course of the detected distributions over time was similar, while a small shift between offline and inline determination could be observed regarding the characteristic t_10_, t_50_ and t_90_-values. However, this is related to the quality of the data sets, since the resolution of the time domain for the offline method is lower in comparison. Additionally, two different inline measuring positions were tested, and an enhanced stability was found for an attachment in the actual die flow channel. This might be related to a smaller disturbance of the flow pattern. As a consequence, a model fit to the data sets obtained in this position lead to a smaller variability of the model parameters. This is important for the monitoring of industrial processes, since deviations from reference parameters have to be identified reliably. The calculated LoQ indicates a high sensitivity of the inline UV/Vis spectroscopy. In addition, a further reduction of the tracer mass by a factor of 5 to 10 should be possible taking into account the ratio of the maximum signal (Figure 6c) to the detected LoQ.

## Symbols

*A*absorbance of light as function of wavelength[-]*B*oBodenstein-number[-]*c*_0_initial concentration[mol·m^−3^]*c*_marker_marker concentration[mol·m^−3^]*cv*coefficient of variation**d*_path_path length of light beam through sample[m]*E*residence time density function[s^−1^]*E*_AD_residence time density function of Axial-Dispersion-Model[s^−1^]*F*cumulative residence time density function[-]*I*transmitted light intensity as function of wavelength[-]*I*_0_basic light intensity as function of wavelength[-]LoQlimit of quantification[-]$\dot{m}$total mass flow of powder inlet[kg·s^−1^]*m*_marker_total marker mass[kg]*s*standard deviation**s*_basic_standard deviation of basic signal**t*time[s]$\bar{t}$mean residence time[s]*t*_i_quantile of the cumulative residence time density function corresponding to the value i[-]*T*rtransmission of light as function of wavelength[-]$\bar{x}$mean value of signal x*${\bar{x}}_{\mathrm{basic}}$mean value of basic signal of the residence time density function[s^−1^]$\varepsilon_{\lambda }$absorbance coefficient as function of wavelength[m^2^·mol^−1^]λwavelength[nm]θdimensionless time[-]* unit depends on measured signal

## Figures and Tables

**Table 1:** Calculated mean value of the characteristic quantiles of the determined residence time density functions in comparison and corresponding standard deviation (*n* = 3).

Method & Measuring Site		*t*_10_ [s]	*t*_50_ [s]	*t*_90_ [s]
HPLC	$\bar{x}\;\pm \;s$	145.4 ± 3.6	221.2 ± 24.1	390.4 ± 44.3
POS 1	$\bar{x}\;\pm \;s$	148.5 ± 7.9	247.4 ± 23.9	483.4 ± 67.6
POS 2	$\bar{x}\;\pm \;s$	152.8 ± 2.2	252.8 ± 23.1	483.5 ± 48.7

**Table 2:** Obtained mean values for the model parameters of the Twin-Dispersion model (TD-model) for all measurement methods and positions with the corresponding standard deviation (*n* = 3).

Method & Measuring Site		*c*_0_ [-]	*B*o_1_ [-]	*B*o_1_ [-]	${\bar{t}}_{1}$ [s]	${\bar{t}}_{2}$ [s]
HPLC	$\bar{x}\;\pm \;s$	0.98 ± 0.009	57.3 ± 38.1	2.75 ± 4.62	131.2 ± 40.9	110.6 ± 167.8
POS 1	$\bar{x}\;\pm \;s$	1.00 ± 0.018	400.5 ± 581.2	1.71 ± 1.59	123.7 ± 17.9	80.8 ± 23.5
POS 2	$\bar{x}\;\pm \;s$	1.00 ± 0.005	150.9 ± 93.8	2.77 ± 1.17	122.1 ± 14.2	93.8 ± 13.0
